# Folate receptor-positive circulating tumor cell count, lymphocyte count and derived neutrophil-to- lymphocyte ratio for diagnosing lung cancer relapse

**DOI:** 10.3389/fonc.2022.1097816

**Published:** 2023-01-19

**Authors:** Huanrong Wang, Lei Liu, Jiaqin Yan, Wang Ma, Yabing Du, Tengfei Zhang

**Affiliations:** Department of Oncology, The First Affiliated Hospital of Zhengzhou University, Zhengzhou, China

**Keywords:** folate receptor-positive circulating tumor cell, lymphopenia, derived neutrophil-to-lymphocyte ratio, lung cancer, relapse, prognosis, first-line treatment

## Abstract

The folate receptor-positive circulating tumor cell (FR^+^-CTC) count can be used to improve the diagnosis rate of lung cancer. The lymphocyte count (LC) and derived neutrophil-to-lymphocyte ratio (dNLR) are involved in inflammatory processes. Whether the FR^+^-CTC count combined with the dNLR or LC is helpful for diagnosing lung cancer recurrence is not clear. Sixty-eight patients who were initially diagnosed with lung cancer and received first-line treatment were included. The clinicopathological characteristics, routine blood examination results and CTC examination results of the patients were collected. The role of the complete blood count and FR^+^-CTC count in lung cancer treatment response and prognosis was analyzed. The FR^+^-CTC count after treatment was significantly correlated with the T stage (*p*=0.005). Multivariate analysis showed that the pathological type and FR^+^-CTC count were independent predictors of disease-or progression-free survival (DFS/PFS) in patients with lung cancer (*p*=0.010 and *p*=0.030, respectively). The FR^+^-CTC count, LC and dNLR predicted the recurrence of lung cancer (sensitivity and specificity of the FR^+^-CTC count, 69.2% and 71.4%; the LC, 50.0% and 88.5%; and the dNLR, 50.0% and 88.1%, respectively). The FR^+^-CTC count combined with the LC or dNLR improved the diagnostic rate of lung cancer recurrence (sensitivity and specificity of the FR^+^-CTC count plus the LC, 53.8% and 90.5%, and the FR^+^-CTC count plus the dNLR, 73.1% and 73.8%, respectively). When these three indicators were combined to predict lung cancer recurrence, the AUC value was 0.817. The FR^+^-CTC count combined with the dNLR and/or LC after treatment can improve the diagnostic rate of lung cancer recurrence. A higher FR^+^-CTC count predicts worse DFS/PFS in patients with lung cancer.

## Introduction

Lung cancer has the highest mortality rate of all cancers, according to the latest statistics from the American Cancer Society (1). According to the pathological type, lung cancer is divided into small cell lung cancer (SCLC) and non-small cell lung cancer (NSCLC), including lung squamous cell carcinoma (LUSC) and lung adenocarcinoma (LUAD). Although SCLC is sensitive to platinum-based chemotherapy agents, it has the highest mortality rate because of its recurrence and progression in a short time (2). Low-dose computed tomography (CT) is the primary means to screen lung cancer at present. In a multicenter study, low-dose CT screening for lung cancer reduced mortality by 20% compared with X-rays during follow-up (3). One study reported that circulating tumor cells (CTCs) were found in the peripheral blood of five patients with chronic obstructive pulmonary disease, and all five CTC-positive patients developed lung cancer during follow-up. The CTC count can be used to detect lung cancer one to four years earlier than chest CT (4). In addition, some studies have shown that identifying tyrosine kinase inhibitor resistance in NSCLC is significantly delayed, with a median lag time of 113 days (range, 45-169 days), based on CT compared with the CTC count (5).

CTCs are released into peripheral blood from primary tumors or during metastasis, and many studies have shown that the CTC count can be used to assist in the diagnosis of breast, gastric, and lung cancers (6–8). Initially, CTCs are epithelial types, but blood is not an ideal place for epithelial cells, so they enter an epithelial-mesenchymal cell change stage; notably, most detection methods cannot detect CTCs that develop this change (9). Folate receptor (FR) mediates folate transport into cells (10). Studies have shown that tumor cells need to absorb a large amount of folic acid for DNA synthesis in the process of proliferation. Hence, FR is highly expressed in most human tumor cells, such as lung cancer, endometrial cancer, and ovarian cancer cells (11–13). By injecting a tumor-specific fluorescent ligand recognition FR *in vivo*, CTCs can be counted by multiphoton fluorescence imaging of surface vessels, and the presence of CTCs can be detected a few weeks before metastatic lesions are observed by other methods (14). Thus, scientists can detect CTCs that undergo epithelial-mesenchymal transition using the ligand-targeted polymerase chain reaction method, which has been approved by the China Food and Drug Administration to detect the number of CTCs (15).

Inflammation is considered to be the main driver of tumorigenesis and progression. The inflammatory cells related to tumor progression are mainly neutrophils (NEs), macrophages and myeloid-derived suppressor cells (16–18). Myelopoiesis in the tumor is associated with defects in myeloid cell differentiation, resulting in the accumulation of immature myeloid cells such as myeloid-derived suppressor cells and NEs in the circulation (16). Lymphocytes are one of the important components of the immune system and are indispensable in the antitumor immune response. A study has shown that a low lymphocyte count (LC) induced by radiation therapy reduces survival in patients with stage III lung cancer (19). In addition, platelets (PLTs) are involved in the development of inflammation; most patients with tumors have higher PLT levels than patients without tumors (20). The neutrophil-to-lymphocyte ratio (NLR) and platelet-to-lymphocyte ratio (PLR) are important in the diagnosis and prognosis of lung cancer and other tumors, such as liver cancer and ovarian cancer (21, 22). In addition, the derived neutrophil-to-lymphocyte ratio (dNLR) is an essential factor in predicting the effect of immunotherapy in NSCLC patients (23). Investigators have demonstrated that the dNLR is associated with the prognosis of metastatic NSCLC, and these results were not affected by treatment plans (24). Thus, we speculated that the folate receptor-positive circulating tumor cell (FR^+^-CTC) count combined with other serological markers, such as the LC, NLR, PLR, and dNLR, during first-line therapy in patients with lung cancer have the potential to be used as biomarkers to predict patient recurrence or progression. Moreover, these indicators are routinely measured in clinical practice. Compared with high invasive and inaccessible markers, it’s more significant to explore these regular hematological tests for tumor diagnosis and prognosis.

## Patients and methods

### Patient recruitment and data collection

In this retrospective study, we enrolled patients who had just been diagnosed with lung cancer and treated between July 2018 and September 2021. We collected their clinicopathological characteristics, including age, sex, pathological type, treatment regimen, and maximum tumor diameter. TNM staging was determined based on the American Joint Committee on Cancer guidelines. Hematological parameters included NE, leukocyte, lymphocyte, PLT and FR^+^-CTC counts. The study followed the guidelines of the Declaration of Helsinki. The study protocol was approved by the Ethics Committee of the First Affiliated Hospital of Zhengzhou University (2002-KY-2021-002).

### Assessments

The inclusion criteria included the following: patients with lung cancer diagnosed by cytology and pathology; patients who had not received any antineoplastic therapy; and complete blood count and FR^+^-CTC count were tested on admission and during treatment. Patients who received treatment only once or had incomplete follow-up data were excluded. Imaging was performed according to RECIST 1.1 to assess stable disease (SD) and progressive disease (PD). Assessments were performed every 1-2 months for the first year of lung cancer ascertainment and every 3-6 months thereafter. Progression-free survival (PFS) referred to the time from randomization to the first occurrence of disease progression or death from any cause. Disease-free survival (DFS) corresponded to the length of time after the operation during which a patient survives with no signs of disease. The follow-up time was up to July 2, 2022.

In this study, the posttreatment hematological indicators of the 68 patients were assessed. For patients who only received surgical treatment and targeted therapy, the indicators were collected within 3 months after treatment, and for patients treated with chemotherapy, indicators were collected at the end of two courses of treatment. The FR^+^-CTC number was detected with the CytoploRare Human Lung Cancer Circulating Tumor Cell Assay Kit (Geno). There are two steps in FR^+^-CTC detection: enrichment by immunomagnetic beads and ligand-targeted polymerase chain reaction to detect and quantify FR^+^-CTCs (25). The FR^+^-CTC count was specified as the number of FR^+^-CTCs detected in a 3 mL blood sample. Because of the heterogeneity of tumors and individual differences in patients, we used a receiver operating characteristic (ROC) curve to find the best optimal value (9.75 FU/3 mL, area under the curve (AUC)=0.744) associated with progression between a high FR^+^-CTC count and a low FR^+^-CTC count. The NLR was defined as the neutrophil count/lymphocyte count, the PLR was defined as the platelet count/lymphocyte group count, and the dNLR was defined as the absolute neutrophil count/white blood cell count-absolute neutrophil count.

### Statistical analysis

The FR^+^-CTC count and other categorical variables were compared with Pearson’s chi-square test. An ROC curve was utilized to analyze the AUC and the corresponding 95% confidence interval (CI), sensitivity, and specificity for each diagnostic variable. The log-rank hazard regression model was used for univariate analysis, and the Kaplan−Meier method was used to draw survival curves. Cox’s proportional hazards regression model was used for multivariate analysis to calculate hazard ratios (HRs) and 95% CIs. Figures in the article were drawn with PRISM 8. P<0.05 was considered statistically significant.

## Results

### Patient characteristics

Sixty-eight patients (35 males and 33 females) who met the criteria were included in our research. The average age at initiation of treatment was 58, with a maximum of 83 and a minimum of 32. We chose the maximum diameter of the tumor to record tumor size. The average tumor size was 2.79 cm, with a minimum of 0.63 cm and a maximum of 7.20 cm. Pathological types included LUAD (n=54), LUSC (n=7) and SCLC (n=7). All patients were initially diagnosed with lung cancer and received first-line treatment in the hospital. Sixteen people received surgical treatment. Fifteen people received chemotherapy with platinum-based single- or double-drug therapy. Three received targeted therapy with oral tyrosine kinase inhibitors. Thirty-four people received two or more combination treatments. By the end of follow-up, 26 patients had disease recurrence or progression. The clinicopathological characteristics of all patients are shown in **Table 1**.

**Table 1:** Clinical and pathological characteristics of the 68 patients.

Characteristics	Total (N=68)
Age (average, range), years	58 (32, 83)
Sex (male/female)	35/33
Pathologic	
LASC	7
LUSC	54
SCLC	7
Tumor size (average, range), cm	2.79(0.63, 7.20)
LC (average, range), ×10^9^/L	1.5(0.49, 3.05)
NE (average, range), ×10^9^/L	5.34(1.65, 10.67)
PLT (average, range), ×10^9^/L	251 (88, 462)
NLR (average, range)	4.01(0.74, 14.28)
PLR (average, range)	183.1(76.0, 578.5)
dNLR (average, range)	2.20(0.49, 5.08)
Treatment	
Surgical only	16
Chemotherapy only	15
Targeted therapy only	3
Combined treatment	34
State of treatment (relapse/no relapse)	26/42

### Association between the FR^+^-CTC count and clinical characteristics

We analyzed the correlation between the posttreatment FR^+^-CTC count and the clinicopathological characteristics of patients. As mentioned, we used 9.75 FU/3 mL as a cutoff value to classify high and low FR^+^-CTC counts. For other clinical characteristics, the average value was used as the dividing line. The FR^+^-CTC count of patients in the T3 and T4 stages was significantly higher than that of patients in the T1 and T2 stages (*p*=0.005). In addition, the CTC value of patients treated with systemic therapy was significantly higher than that of patients treated with surgery (*p*=0.019). However, other indicators were not significantly different from the FR^+^-CTC count ​​(**Table 2**).

**Table 2:** Correlation between the FR^+^-CTC count and clinical characteristics.

Characteristics	CTC expression level		*χ2*	*p*	Characteristics	CTC expression level		*χ2*	*p*
	**Low (n=38)**	**High(n=30)**				**Low (n=38)**	**High(n=30)**		
**Age(years)**			1.36	0.243	**Clinical stage**			3.51	0.061
<58	18(47.4%)	10(33.3%)			I	20(52.6%)	9(30.0%)		
≥58	20(52.6%)	20(66.7%)			II+III+IV	18(47.4%)	21(70.0%)		
**Sex**			0.5	0.481	**PLT (×10^9^/L)**			1.56	0.211
Male	21(55.2%)	14(46.6%)			<251	21(55.3%)	12(40.0%)		
Female	17(44.8%)	16(53.4%)			≥251	17(44.7%)	18(60.0%)		
**Tumor size(cm)**			0.68	0.411	**NE(×10^9^/L)**			0.002	0.965
<2.79	19(50.0%)	18(60.0%)			<5.34	23(60.5%)	18(60.0%)		
≥2.79	19(50.0%)	12(40.0%)			≥5.34	15(39.5%)	12(40.0%)		
**Pathologic type**			3.76	0.053	**NLR**			0.14	0.712
NSCLC	37(97.4%)	24(80.0%)			<4.01	25(65.8%)	21(70.0%)		
SCLC	1(2.6%)	6(20.0%)			≥4.01	13(34.2%)	9(30.0%)		
**Treatment**			5.46	**0.019**	**PLR**			1.09	0.297
Surgery	13(34.2%)	3(10.0%)			<183.1	25(65.8%)	16(53.3%)		
Systemic therapy	25(65.8%)	27(90.0%)			≥183.1	13(34.2%)	14(46.7%)		
**T classification**			7.84	**0.005**	**dNLR**			1.19	0.276
T1+T2	33(86.8%)	17(56.7%)			<2.2	24(63.2%)	15(50.0%)		
T3+T4	5(13.2%)	13(43.3%)			≥2.2	14(36.8%)	15(50.0%)		
**N classification**			1.73	0.189	**LC(×10^9^/L)**			0.19	0.666
N0	25(65.8%)	15(50.0%)			<1.5	17(44.7%)	15(50.0%)		
N1+N2+N3	13(34.2%)	15(50.0%)			≥1.5	21(55.3%)	15(50.0%)		
**Metastasis tatus**			0.66	0.416					
Yes	31(81.6%)	22(73.3%)							
No	7(18.4%)	8(26.7%)							

Values in bold mean p<0.05.

### Survival analysis

To study the factors affecting the DFS/PFS of lung cancer patients in depth, we included indicators such as sex, age, pathological type, tumor size, TNM stage, treatment regimen and FR^+^-CTC count, NE count, LC, PLT count, dNLR, PLR, and NLR after treatment. Univariate analysis showed that pathological type, T classification, clinical stage, treatment regimen, FR^+^-CTC count, LC, dNLR, and NLR were factors influencing PFS (**Table 3**).

**Table 3:** Univariate and multivariate analyses of disease- or progression-free survival (N = 68, 26 progression events).

	DFS/PFS					
	Univariate Analysis			Multivariate Analysis		
Characteristic	HR	95% CI	*p*	HR	95% CI	*p*
Male (vs. female)	0.570	0.258-1.259	0.164	0.362	0.122-1.073	0.067
Age (per year)	2.267	0.952-5.397	0.064	0.784	0.214-2.872	0.713
SCLC (vs. NSCLC)	12.482	4.440-35.095	**0.000**	5.516	1.510-20.140	**0.010**
Tumor size(cm) ≥2.79(vs. <2.79)	1.596	0.734-3.470	0.238			
T3+T4 (vs. T1+T2)	2.695	1.231-5.899	**0.013**	0.612	0.142-2.645	0.511
Lymph node metastatic yes (vs. no)	1.937	0.893-4.201	0.094	0.816	0.228-2.918	0.755
Metastasis yes (vs. no)	1.040	0.416-2.597	0.933			
Clinical stage others (vs. I)	3.014	1.202-7.554	**0.019**	3.070	0.457-20.634	0.249
Systemic therapy (vs. surgery)	8.476	1.147-62.611	**0.036**	2.986	0.351-25.417	0.317
NE(x10^9^/L) ≥6.65 (vs. <6.65)	0.616	0.231-1.641	0.333			
LC(x10^9^/L) ≥1.72 (vs. <1.72)	0.206	0.062-0.686	**0.010**	0.383	0.101-1.455	0.159
PLT(x10^9^/L) ≥190 (vs. <190)	2.192	0.656-7.314	0.202			
dNLR≥2.46 (vs. <2.46)	5.105	2.235-11.661	**<0.001**	1.852	0.672-5.100	0.233
PLR≥171.5 (vs. <171.5)	2.149	0.972-4.751	0.059	1.965	0.673-5.738	0.217
NLR≥1.93 (vs. <1.93)	4.388	1.035-18.609	**0.045**	2.220	0.420-11.725	0.348
FR^+^-CTC(Fu/3ml) ≥9.75(vs. <9.75)	3.861	1.674-8.908	**0.002**	3.039	1.111-8.310	**0.030**

Values in bold mean p<0.05.

**Figure 1:**
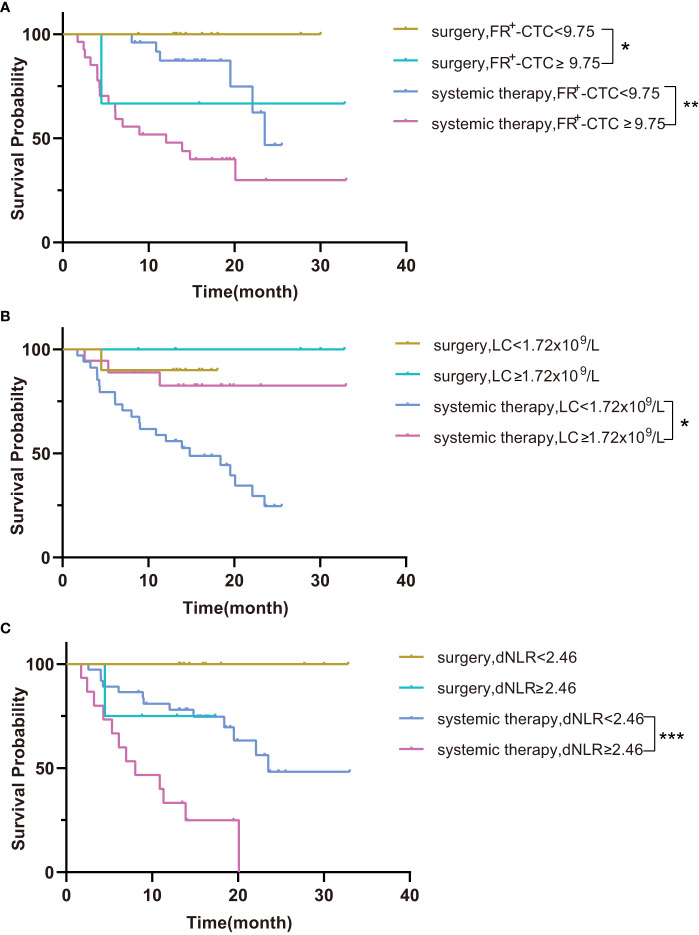
**(A)** Kaplan–Meier curves for survival probability according to the FR^+^-CTC count between surgery and systemic therapy. **(B)** Kaplan–Meier curves for survival probability according to the LC between surgery and systemic therapy. **(C)** Kaplan–Meier curves for survival probability according to the dNLR between surgery and systemic therapy. *P < 0.05, **P < 0.01, ***P < 0.001.

**Figure 2:**
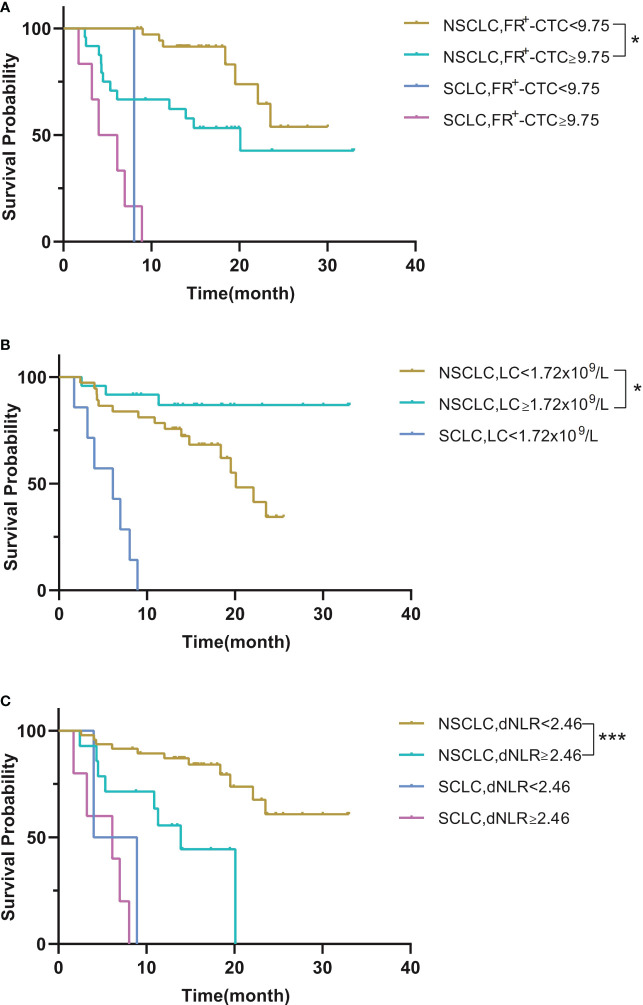
**(A)** Kaplan–Meier curves for survival probability according to the FR^+^-CTC count between NSCLC and SCLC. **(B)** Kaplan–Meier curves for survival probability according to the LC between NSCLC and SCLC. **(C)** Kaplan–Meier curves for survival probability according to the dNLR between NSCLC and SCLC. *P < 0.05, **P < 0.01, ***P < 0.001.

Next, we performed survival analysis of different groups stratified by the first-line treatment regimen and pathological classification. For patients in the surgery group, the median disease-free survival (mDFS) of patients with a low FR^+^-CTC count was significantly longer than that of patients with a high FR^+^-CTC count (*p*=0.046, **Figure 1A**, **Table 4A**). According to the cutoff value of the ROC curve, the LC and dNLR were used to divide patients into high and low groups, and we found that there was no significant difference in DFS between the two groups. For the systemic treatment group, we found that the median progression-free survival (mPFS) of patients with a low FR^+^-CTC count was significantly longer than that of patients with a high FR^+^-CTC count (23.53 months vs. 12.03 months, *p*=0.003, **Figure 1A**, **Table 4A**). Patients with a high LC were more likely than those with a low LC to experience a significantly prolonged mPFS (not reach (NR) vs. 14.8 months, *p*=0.029, **Figure 1B**, **Table 4A**). Patients with low dNLR values ​​had a significantly longer mPFS than patients with high dNLR values ​​(23.53 months vs. 8.033 months, *p*<0.001, **Figure 1C**, **Table 4A**). There was no significant difference in the prognosis of high NLR and low NLR in different treatment groups (**Supplementary Figure 1**). In addition, we divided patients into two groups according to NSCLC and SCLC. For the NSCLC group, the mDFS/mPFS of those with a low FR^+^-CTC count was significantly longer than that of patients with a high FR^+^-CTC count (NR vs. 20.1 months, *p*=0.014, **Figure 2A**, **Table 4B**). Patients with a low LC had a significantly shorter mDFS/mPFS than patients with a high LC (20.10 months vs. NR, *p*=0.029, **Figure 2B**, **Table 4B**), and patients with low dNLR values ​​had a significantly longer mDFS/mPFS than patients with high dNLR values (NR vs. 13.9 months, *p*<0.001, **Figure 2C**, **Table 4B**). For the SCLC group, there was no significant difference in mPFS based on different blood markers (**Figure 2**, **Table 4B**).

Table 4ASurvival probability according to the FR^+^-CTC count, LC and dNLR between surgery and systemic therapy.

**Table:** 

		Median (months)	*p*
FR^+^-CTC(FU/3ml) <9.75 vs. ≥9.75	surgery	NR	0.046
	systemic therapy	23.53vs.12.03	0.003
LC(x10^9^/L) <1.72 vs.≥1.72	surgery	NR	0.480
	systemic therapy	14.8vs. NR	0.029
dNLR <2.46 vs.≥2.46	surgery	NR	0.097
	systemic therapy	23.53vs.8.033	<0.001

Table 4BSurvival probability according to the FR+-CTC count, LC and dNLR between NSCLC and SCLC.

**Table:** 

		Median (months)	*p*
FR^+^-CTC(FU/3ml) <9.75 vs. ≥9.75	NSCLC	NRvs.20.1	0.014
	SCLC	8.033vs.5.067	0.569
LC(x10^9^/L)<1.72 vs.≥1.72	NSCLC	20.1vs.NR	0.029
	SCLC	6.133vs.-	–
dNLR <2.46 vs.≥2.46	NSCLC	NRvs.13.9	<0.001
	SCLC	6.467vs.6.133	0.338

According to the results of univariate analysis, variables with *p*<0.2 were included in multivariate analysis. We found that the FR^+^-CTC count and pathological type were independent predictors of DFS/PFS (**Table 3**).

### ROC analysis of the FR^+^-CTC count, LC and dNLR and the diagnostic yield of joint diagnostic models

**Figure 3:**
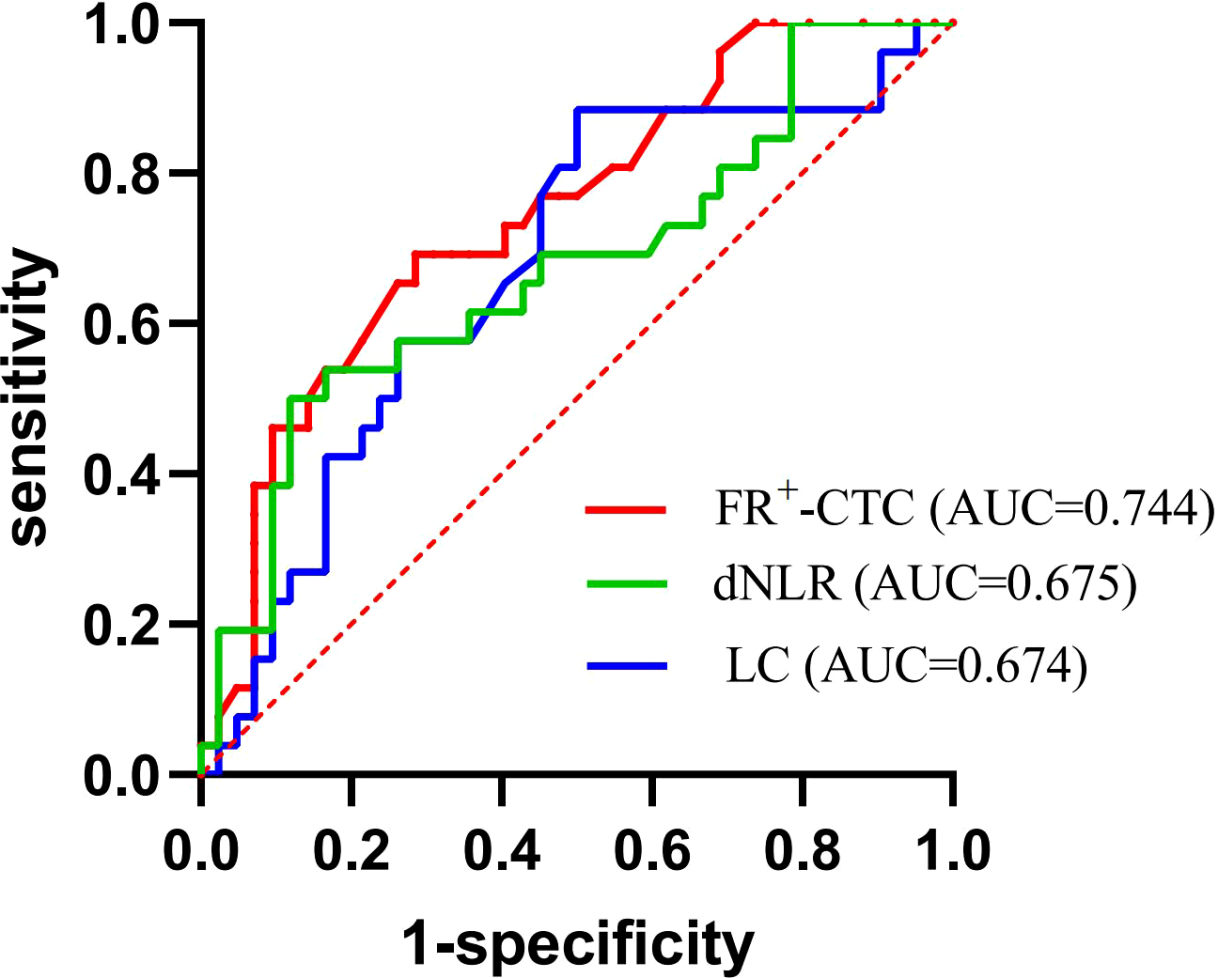
ROC curves of the FR^+^-CTC count, dNLR, and LC in the diagnosis of lung cancer recurrence.

Next, we investigated whether the FR^+^-CTC count, NE count, LC, PLT count, dNLR, PLR and NLR could predict recurrence or progression after first-line treatment of lung cancer. According to our analysis, the optimal cutoff value of the FR^+^-CTC count for differentiating disease recurrence from disease stabilization was 9.75 FU/3 mL, with a sensitivity of 69.2% and a specificity of 71.4% (AUC=0.744). Similarly, the LC and dNLR significantly differentiated disease recurrence (sensitivity=50.0%, specificity=88.5%, AUC= 0.674 and sensitivity=50.0%, specificity=88.1%, AUC=0.675, respectively; **Figure 3**, **Table 5**).

**Table 5:** The diagnostic efficiency of models in patients with lung cancer.

Diagnostic Group	AUC	95% CI	Sensitivity (%)	Specificity (%)	*p*
FR^+^-CTC	0.744	0.625-0.863	69.2	71.4	0.001
LC	0.674	0.540-0.807	50.0	88.5	0.017
dNLR	0.675	0.540-0.811	50.0	88.1	0.016
FR^+^-CTC + LC	0.759	0.638-0.881	53.8	90.5	<0.001
FR^+^-CTC + dNLR	0.762	0.645-0.879	73.1	73.8	<0.001
FR^+^-CTC + LC +dNLR	0.817	0.715-0.919	73.1	78.6	<0.001

**Figure 4:**
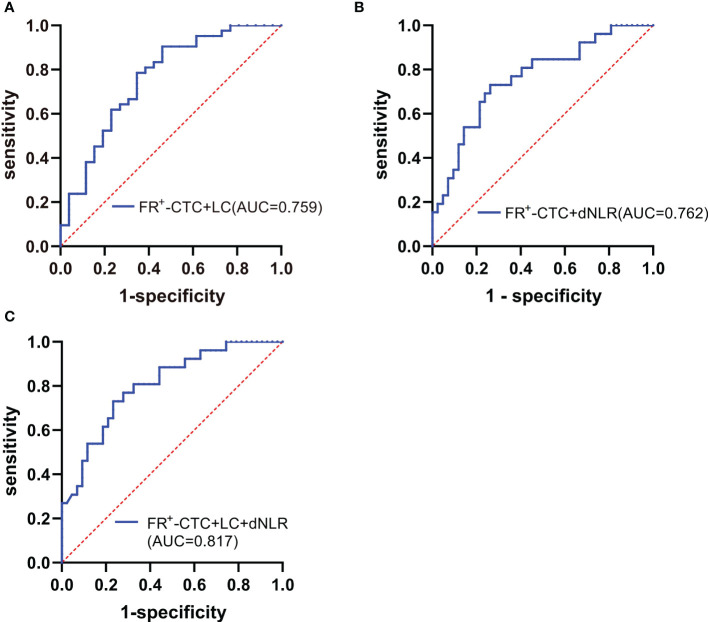
**(A)** ROC curve of the FR^+^-CTC count plus LC in the diagnosis of lung cancer recurrence. **(B)** ROC curve of the FR^+^-CTC count plus dNLR in the diagnosis of lung cancer recurrence. **(C)** ROC curve of the FR^+^-CTC count combined with the LC and dNLR in the diagnosis of lung cancer recurrence.

**Figure 5:**
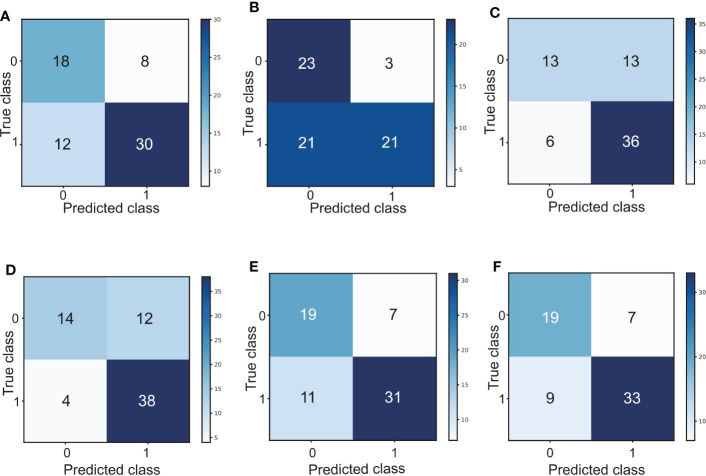
**(A)** Confusion matrix for predicting lung cancer relapse in patients based on the FR^+^-CTC count. **(B)** Confusion matrix for predicting lung cancer relapse in patients based on the LC. **(C)** Confusion matrix for predicting lung cancer relapse in patients based on the dNLR. **(D)** Confusion matrix of the FR^+^-CTC count combined with the LC to predict lung cancer relapse in patients. **(E)** Confusion matrix of the FR^+^-CTC count combined with the dNLR to predict lung cancer relapse in patients. **(F)** Confusion matrix of the FR^+^-CTC count combined with the LC and dNLR to predict lung cancer relapse in patients. 0: relapse; 1: no relapse.

Interestingly, by combining the FR^+^-CTC count with the LC or dNLR, we found that the combined AUC was more significant than the AUC of either one alone (sensitivity and specificity of the FR^+^-CTC count plus LC, 53.8% and 90.5%, AUC=0.759, **Figure 4A**, **Table 5**; sensitivity and specificity of the FR^+^-CTC count plus the dNLR, 73.1% and 73.8%, AUC=0.762, **Figure 4B**, **Table 5**). When these three indicators were combined to diagnose lung cancer recurrence, the AUC value was higher than when any two were combined (sensitivity=73.1%, specificity= 78.6%, AUC=0.817, **Figure 4C**, **Table 5**). The above results indicated that the FR^+^-CTC count combined with the LC and/or dNLR could improve the diagnosis rate of relapse in lung cancer. To present the overall predictive effect of the data, we divided all patients into two categories: relapse and no relapse. The confusion matrix for each hematological marker for predicting lung cancer relapse in patients is shown in **Figure 5**.

We found that the NE count, PLT count, PLR and NLR were not very useful for diagnosing lung cancer recurrence, so we do not show them here (**Supplementary Figure 2**).

## Discussion

Although oncological research on lung cancer has been progressing yearly, recurrence or progression during treatment is still the most direct cause of lung cancer treatment failure. Over the past 20 years, researchers have demonstrated that CTCs play an essential role in the diagnosis and prognosis of malignant tumors (26, 27). However, the search for an efficient method to isolate and detect CTCs remains controversial, and oncologists are just beginning to understand how the information obtained from CTC detection and analysis can be used in patient care. In this study, we designed a diagnosis-treat-relapse model to investigate the significance of CTCs and other hematological markers for diagnosing lung cancer recurrence. We aimed to use these clinical hematological markers to detect lung cancer recurrence and adjust the treatment plan in time to bring the greatest clinical benefit to the patients.

Researchers have reported that the FR^+^-CTC count can predict prognosis in breast cancer, gastrointestinal tumors, melanoma and prostate cancer (6, 28–31). A prospective, single-center clinical study showed that a lower FR^+^-CTC count could be a predictor of first-line chemotherapy in SCLC patients (HR=0.656, *p*=0.049) (15). Hiltermann et al. found that the CTC count after the first cycle of chemotherapy predicted overall survival (HR=5.7, *p*=0.004) (32). All of the above findings suggest that the CTC count has important value in lung cancer predictions. In this study, we demonstrated a significant correlation between CTC levels and T stage. Patients with a high T stage had higher FR^+^-CTC count after treatment than those with a low T stage, indicating that similar to T stage, CTC levels may negatively affect patient survival. In addition, since patients with early-stage lung cancer received surgical treatment, while patients with advanced lung cancer received systemic treatment, patients treated with surgery had significantly lower CTCs than patients treated with systemic treatment.

Early recurrence in patients with resectable NSCLC ranges from 10-19% (33, 34). Although imaging tests have been used as the standard for tumor response assessment, they can only be used to detect space-occupying lesions and not minimal residual disease. A study of 26 early-stage lung cancer patients showed that after standard surgical treatment, the descending slope and daily decline in CTCs in recurrent lung cancer patients were lower than those in nonrecurring patients (35). We found that patients with systemic treatment and high FR^+^-CTC values had the fastest decline in the survival curve and the worst prognosis. In addition, we also found that within 22 months after treatment, the mortality rate of patients with early-stage lung cancer with high postoperative FR^+^-CTC was even higher than that of patients with advanced lung cancer with low FR^+^-CTC. The survival curves of the low LC group and high dNLR group of advanced lung cancer decreased rapidly, and the prognosis was poor. Among the patients with a higher dNLR after systemic treatment, the last patient developed progression by the end of follow-up, and PFS was 20.1 months. This finding suggests that patients with higher FR^+^-CTC count after treatment may have occult metastases and that systemic therapy is necessary for better disease control.

Investigators have previously reported that the FR^+^-CTC count can be used to diagnose stage I LUAD and LUSC patients with sensitivities of 66.7% and 69.2%, respectively (25). A recent study showed that in SCLC patients, a lower lymphocyte count was associated with worse overall survival (17.4 vs. 15.7 months, *p*=0.029) (36). A study of 211 patients who received first-line pembrolizumab treatment demonstrated that a dNLR < 2.6 significantly prolonged patients’ mPFS (10.4 vs. 3.4 months, *p*< 0.001) (37). To investigate the diagnostic value of the FR^+^-CTC count, dNLR, LC, PLR and NLR in recurrence, we performed ROC curve analysis of individual and combined indicators. We found that the FR^+^-CTC count, dNLR and LC could be used as indicators for diagnosing lung cancer recurrence. In addition, when the FR^+^-CTC count was combined with the dNLR and/or LC, it had a larger AUC than the indicators alone. This demonstrates that the combination of the FR^+^-CTC count and dNLR and/or LC can improve the diagnosis rate at the time of recurrence of lung cancer.

Our study supports the evidence proposed by Chen et al. (38). This is the first study to use the FR^+^-CTC count combined with the dNLR and/or LC to diagnose relapse rates after first-line therapy for lung cancer.

There are several limitations in this study. The research data were mainly entered manually, although no clinicopathological, hematological or survival data were missing. In addition, we established a cutoff value for the FR^+^-CTC count based on our previous results rather than using a positive/negative threshold for the FR^+^-CTC count (8.7 FU/3 mL positive) (39). However, this count was verified as appropriate for diagnosing patients who relapsed during treatment. Furthermore, because the FR^+^-CTC examination was not included in the routine examination of lung cancer patients, the number of SCLC patients included in this study was relatively small, and most of them had high FR^+^-CTC counts. The impact of FR^+^-CTC count on the survival rate of SCLC patients may not have been fully analyzed. However, SCLC patients with a higher FR^+^-CTC count ​​had a faster decline in the survival curve and a lower survival rate, which is also consistent with clinical significance.

In conclusion, the FR^+^-CTC count, dNLR and LC are useful biomarkers. A high posttreatment FR^+^-CTC count, dNLR and LC are indicators that predict whether a patient will progress, and the combined indicators had a better prediction effect. In addition, a higher FR^+^-CTC count was significantly associated with shorter patient survival.

## Data availability statement

The raw data supporting the conclusions of this article will bemade available by emailing corresponding authors.

## Ethics statement

The studies involving human participants were reviewed and approved by Ethics Committee of the First Affiliated Hospital of Zhengzhou University. Written informed consent for participation was not required for this study in accordance with the national legislation and the institutional requirements.

## Author contributions

HW was responsible for conceptualization, methodology, software, formal analysis, original draft preparation, review and editing; LL was responsible for validation, formal analysis, review and editing; JY and WM were responsible for supervision; YD was responsible for formal analysis, resources, investigation, supervision, review and editing; TZ was responsible for formal analysis, data curation, review and editing, supervision, project administration and funding acquisition. All authors contributed to the article and approved the submitted version.

## Supplementary material

Supplementary Figure 1 Kaplan–Meier curves for survival probability according to the NLR between surgery and systemic therapy.

Supplementary Figure 2 ROC curves of the NE count, PLT count, NLR and PLR in the diagnosis of lung cancer recurrence.
