# Supplementary figures and images for: Folate receptor-positive circulating tumor cell count, lymphocyte count and derived neutrophil-to- lymphocyte ratio for diagnosing lung cancer relapse
Source: Front Oncol. 2023 Jan 19;12:1097816. doi: 10.3389/fonc.2022.1097816 (PMC9893416; doi:10.3389/fonc.2022.1097816)

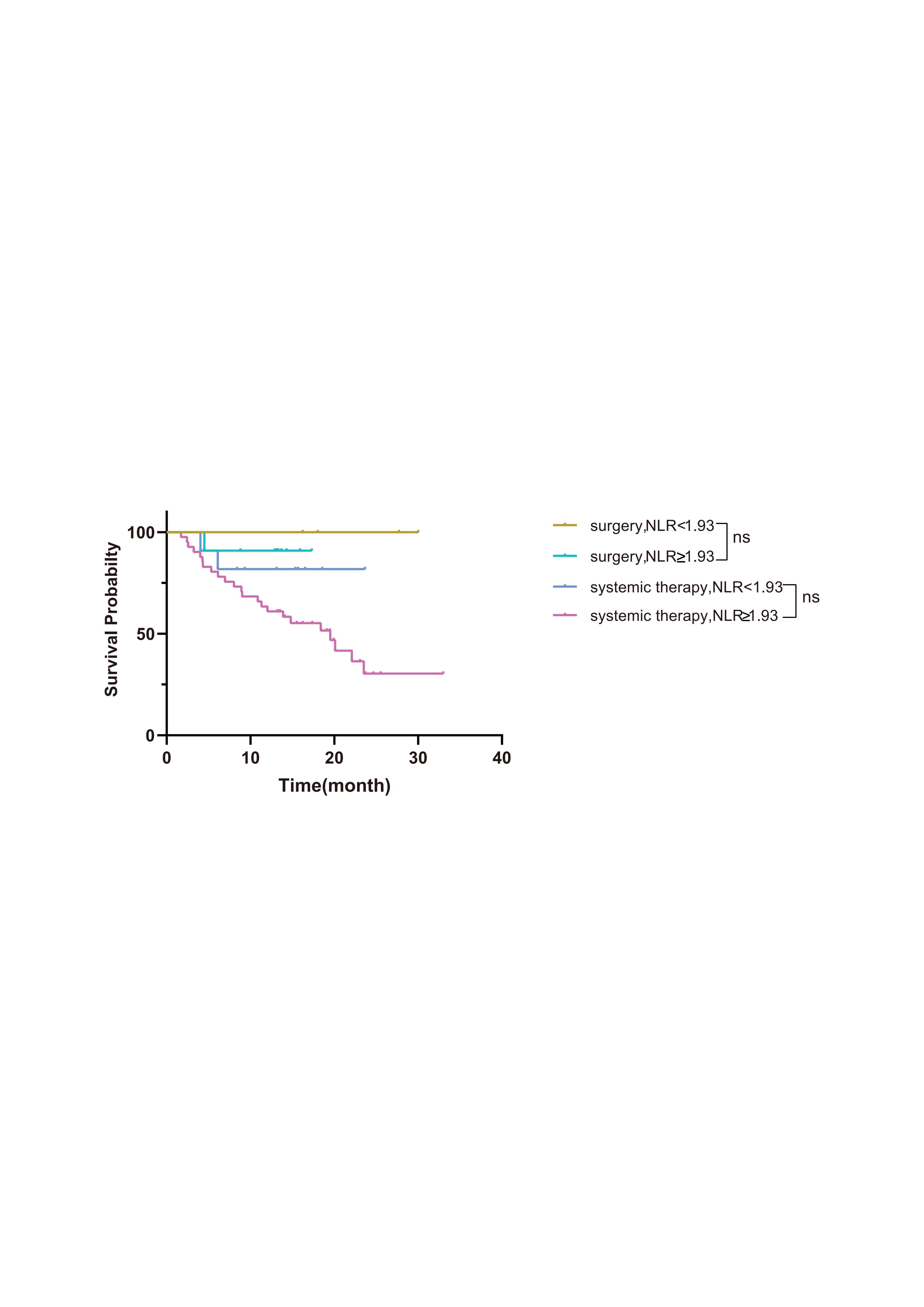

Supplement: Supplementary Figure 1 — Kaplan–Meier curves for survival probability according to the NLR between surgery and systemic therapy. [file Image_1.jpeg]

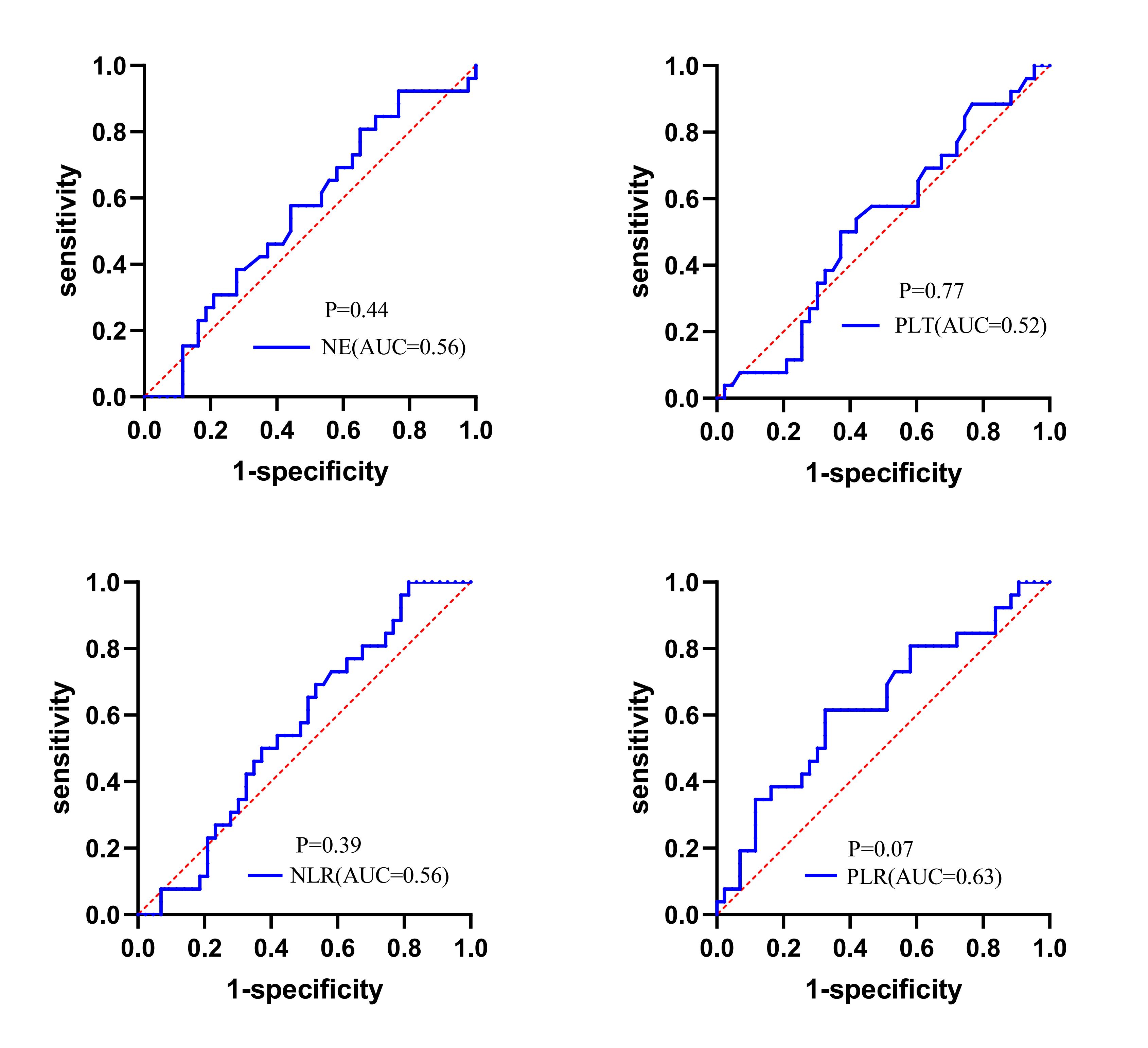

Supplement: Supplementary Figure 2 — ROC curves of the NE count, PLT count, NLR and PLR in the diagnosis of lung cancer recurrence. [file Image_2.jpeg]
